# CETSA MS Profiling for a Comparative Assessment of FDA-Approved Antivirals Repurposed for COVID-19 Therapy Identifies TRIP13 as a Remdesivir Off-Target

**DOI:** 10.1177/2472555220973597

**Published:** 2020-11-18

**Authors:** Tomas Friman, Alexey Chernobrovkin, Daniel Martinez Molina, Laurence Arnold

**Affiliations:** 1 Pelago Bioscience AB, Solna, Sweden

**Keywords:** CETSA MS, COVID-19, antivirals, TRIP13, target engagement

## Abstract

The reuse of preexisting small molecules for a novel emerging disease threat is a rapid measure to discover unknown applications for previously validated therapies. A pertinent and recent example where such a strategy could be employed is in the fight against coronavirus disease 2019 (COVID-19). Therapies designed or discovered to target viral proteins also have off-target effects on the host proteome when employed in a complex physiological environment. This study aims to assess these host cell targets for a panel of FDA-approved antiviral compounds including remdesivir, using the cellular thermal shift assay (CETSA) coupled with mass spectrometry (CETSA MS) in noninfected cells. CETSA MS is a powerful method to delineate direct and indirect interactions between small molecules and protein targets in intact cells. Biologically active compounds can induce changes in thermal stability, in their primary binding partners, and in proteins that in turn interact with the direct targets. Such engagement of host targets by antiviral drugs may contribute to the clinical effect against the virus but can also constitute a liability. We present here a comparative study of CETSA molecular target engagement fingerprints of antiviral drugs to better understand the link between off-targets and efficacy.

## Introduction

The coronavirus disease 2019 (COVID-19) pandemic has seen a significant worldwide effort to reuse or repurpose preexisting therapies in order to combat the emerging viral threat. There have been numerous studies reported using a variety of technologies in an effort to screen panels of prevalidated molecules, many repurposed from viral therapies.^1–5^ These studies are conducted in the hope that efficacy against the SARS-CoV-2 virus may be discovered, while avoiding the lengthy yet essential drug discovery pipeline that even with modern standards typically takes several years from hit and target identification to reach clinical testing of a lead candidate drug molecule.^6^

Utilising a preexisting molecule has a significantly lower risk than rapidly developing novel chemistry, since it has already successfully navigated the prerequisite safety and toxicologic testing for use in humans. However, the original purpose of the small molecule may have undescribed off-target effects that are deemed to be tolerable when weighed against therapeutic benefit. These effects, potentially caused by drug–protein interactions, are often poorly understood.^7^

For example, many antiviral compounds are structural analogs of nucleoside triphosphates that have diverse biological properties and therapeutic consequences since nucleotides have an essential role in virtually all biological processes.^8^ Therefore, given the abundance of nucleotide interacting proteins in the host cell, off-target interacting proteins, or an imbalance of the cellular nucleotide pool, would be an expected consequence of utilizing nucleotide analogs in therapy.^9^

The persistent and fundamental problem of host off-target effects arises from using a molecule to disrupt viral biology, while simultaneously exposing the host biology to the same chemical challenge. Methods to describe the severity of hitting off targets rely upon in vitro and in vivo assessment or the presentation of a phenotype that can be assessed as to whether it is acceptable or not. However, this requires knowledge and the ability to measure nonintended target biology. For example, remdesivir is known to have an efficacy of 100 nM (IC_50_) for the viral polymerase, its intended target, and is 500-fold less efficacious against human polymerases.^10^ It has previously not been established which other proteins it may interact with and or whether these potential interactions would elicit a response with a measurable output using conventional means.

In light of this, traditional off-target investigation relies on known functions or activities that, as a prerequisite, require the host proteins responsible for these activities to be studied in a biased manner. Methods that are independent of activity and report on compound interaction against the entire proteome in an unbiased way have only in recent years been established.^11,12^ The cellular thermal shift assay (CETSA) is a powerful technique to detect protein–ligand interactions in cells.^13^ Coupled with mass spectrometry (MS) as a readout, CETSA MS is a technique employed in the identification of off-target effects in proteome-wide studies observing the thermal stabilization or destabilization of endogenous proteins and downstream effects after matrix and compound incubation. The method is increasingly being employed both in mechanism of action (MoA) studies and to identify primary and off-targets of candidate drug molecules, for example, quinine and drug target interactions in *Plasmodium falciparum*.^14,15^ In this study, we screened a panel of drugs using the CETSA MS format on HepG2 cells to identify host proteins as hopeful starting points for further research and possible inroads into the improvement or development of fortuitous therapies for SARS-CoV-2 infection.

Given the intense global interest in searching for a viable therapy combined with the wide accessibility to information sources and even raw data, efforts from a wide variety of groups have been well documented in both the scientific and nonscientific media. The inclusion of compounds for this study was directed around prominent molecules discussed in the literature and adopted for clinical trials in the earlier phases of the worldwide pandemic, namely, remdesivir and hydroxychloroquine. The study was bolstered by the addition of other compounds repurposed from a variety of antiviral classes, including retroviral reverse transcriptase and protease inhibitors that were available for expeditious purchase from commercial sources.^16–19^

This study investigates compound effects on uninfected whole HepG2 cells. Understanding how the molecule reacts in an environment containing both viral and host cell proteins is not beyond the technique, but is outside of the capacity and scope for this study, which was completed utilizing a preexisting platform with a per-compound acquisition time of approximately 6 h.

## Materials and Methods

### Cell Culture

The human cell line HepG2 was procured from the American Type Culture Collection and cultured until 70% confluency in collagen-coated flasks. The cells were cultured in Dulbecco’s modified Eagle’s medium without phenol red (DMEM/F12, Thermo Fisher, Sweden) supplemented with 10% fetal bovine serum (FBS; Thermo Fisher), 5 mM sodium pyruvate (Thermo Fisher), 1× nonessential amino acids (NEAAs; Thermo Fisher), and penicillin-streptomycin (PEST; Thermo Fisher). Cells were detached using 5 mL of Tryp-LE (Thermo Fisher Scientific), pelleted, washed with Hank’s Balanced Salt Solution (HBSS; Thermo Fisher Scientific), and pelleted again. Cell viability was measured with trypan blue exclusion, and cells with a viability above 90% were used for these experiments. Cell pellets were resuspended in medium-free incubation buffer (20 mM HEPES, 138 mM NaCl, 5 mM KCl, 2 mM CaCl_2_, 1 mM MgCl_2_, pH 7.4) for further use as a 2× cell suspension.

### Compound Handling

All compounds were acquired from commercial sources as powder stocks and reconstituted in DMSO or aqueous buffer dependent on manufacturer-recommended solubility values. The DMSO concentration was normalized across all samples to a final concentration of 1% v/v.

### Compressed CETSA MS Experiment

The cell suspension was divided into 30 aliquots (22 test compounds, 2× methotrexate [first positive control], 2× vincristine [second positive control], and 4× negative/vehicle controls) in 1.5 mL tubes and mixed with an equal volume of either the test compound or controls at 2× the final concentration in the experimental buffer. The resulting final concentration of the compounds was 30 µM; 1% DMSO was used as a vehicle control. Incubations were performed for 60 min at 37 °C with end-over-end rotation.

Each of the treated cell suspensions was further divided into 12 aliquots that were all subjected to a heat challenge for 3 min, each at a different temperature between 44 and 66 °C. After heating, all temperature points for each test condition were pooled to generate 32 individual (compressed) samples.

Precipitated proteins were pelleted by centrifugation at 30,000*g* for 20 min, and supernatants constituting the soluble protein fraction were kept for further analysis.

The experiment was performed over three independent biological replicates.

### Protein Digestion

The total protein concentration of the soluble fractions was measured by Lowry DC assay (Bio-Rad, Hercules, CA). From each soluble fraction, a volume containing an equivalent of 20 µg of total protein was taken for further sample preparation.

Samples were subjected to reduction and denaturation with tris(2-carboxyethyl)phosphine (TCEP; Bond-breaker, Thermo Scientific), followed by alkylation with chloroacetamide. Proteins were digested with Lysyl-C (Wako Chemicals, Germany) and trypsin (Trypsin Gold, Promega, Sweden).

### TMT Labeling of Peptides

After complete digestion had been confirmed by nano-liquid chromatography tandem mass spectrometry (LC-MS/MS), samples were labeled with 16-plex Tandem Mass Tag reagents (TMTpro, Thermo Scientific) according to the manufacturer’s protocol.

Labeling reactions were quenched by addition of a primary amine buffer, and the test concentrations and room temperature control samples were combined into TMT16-plex sets such that each TMT16-multiplex set contained 12 test compounds, 2 positive control samples (methotrexate + vincristine), and 2 negative controls (1% DMSO). The labeled samples were subsequently acidified and desalted using polymeric reversed-phase chromatography (Oasis Waters, Milford, MA). LC-MS-grade liquids and low-protein binding tubes were used throughout the purification. Samples were dried using a centrifugal evaporator.

### LC-MS/MS Analysis

For each TMT16-multiplex set, the dried labeled sample was dissolved in 20 mM ammonium hydroxide (pH 10.8) and subjected to reversed-phase high-pH fractionation using an Agilent 1260 Bioinert HPLC system (Agilent Technologies) over a 1.5 × 150 mm C18 column (XBridge Peptide BEH C18, 300 Å, 3.5 µm particle size; Waters Corporation, Milford). Peptide elution was monitored by UV absorbance at 215 nm, and fractions were collected every 30 s into polypropylene plates. The 60 fractions covering the peptide elution range were evaporated to dryness, ready for LC-MS/MS analysis.

From the fractions collected, 30 pooled fractions were analyzed by high-resolution nano-LC-MS/MS on Q-Exactive HF-X Orbitrap mass spectrometers (Thermo Scientific) coupled with high-performance nano-LC systems (Ultimate 3000 RSLC Nano, Thermo Scientific).

MS/MS data were collected using higher-energy collisional dissociation (HCD), and full MS data were collected using a resolution of 120 K with an automatic gain control (AGC) target of 3e6 over the *m*/*z* range 375–1500. The top 15 most abundant precursors were isolated using a 1.4 Da isolation window and fragmented at normalized collision energy values of 35. The MS/MS spectra (45 K resolution) were allowed a maximal injection time of 120 ms with an AGC target of 1e5 to avoid coalescence. The dynamic exclusion duration was 30 s.

### Protein Identification and Quantification

Protein identification was performed by a database search against 95,607 human protein sequences in Uniprot (UP000005640, download date: Oct 21, 2019) using the Sequest HT algorithm as implemented in the Proteome Discoverer 2.4 software package. Data were recalibrated using the recalibration function in Proteome Discoverer 2.4, and final search tolerance settings included mass accuracies of 10 ppm and 50 mDa for precursor and fragment ions, respectively. A maximum of two missed cleavage sites were allowed using fully tryptic cleavage enzyme specificity (K, R, no P). Dynamic modifications were oxidation of Met, and deamidation of Asn and Gln. Dynamic modification of protein N-termini by acetylation was also allowed. Carbamidomethylation of Cys, TMTpro modification of lysine, and peptide N-termini were set as static modifications.

For protein identification, validation was done at the peptide–spectrum–match (PSM) level using the following acceptance criteria: 1% false discovery rate determined by Percolator scoring based on Q value, rank 1 peptides only.

For quantification, a maximum co-isolation of 50% was allowed. Reporter ion integration was done at 20 ppm tolerance, and the integration result was verified by manual inspection to ensure the tolerance setting was applicable. For individual spectra, an average reporter ion signal-to-noise ratio of >20 was required. Only unique or razor peptides were used for protein quantification.

### Data Analysis

Quantitative results were exported from Proteome Discoverer as tab-separated files and analyzed using R version 4.0.2 software. Protein intensities in each TMT channel were log2-transformed and normalized by subtracting the median value for each TMT sample and each TMT channel (column-wise normalization). For each protein and each compound, thermal stability changes were assessed by comparing normalized log2-transformed intensities to the DMSO-treated control using a moderated *t* test implemented in “limma” R package version 3.44.1.^20^

### CETSA with Western Blot Detection

HepG2 cells were cultured, harvested, and washed as previously mentioned. For the intact cell study, the cells at a concentration of 10 million/mL in HBSS were aliquoted and incubated with either 30 µM remdesivir or volume-matched vehicle control (DMSO). The samples were incubated at 37 °C for 60 min with gentle mixing. The suspensions were further aliquoted and subjected individually to a 12-temperature heat gradient between 37 and 63 °C for 3 min and snap frozen in liquid nitrogen. The samples were lysed by three rounds of freeze–thaw and the insoluble fraction pelleted by centrifugation at 20,000*g* for 20 min at 4 °C. Soluble proteins were resolved using NuPAGE Novex Bis-Tris 4%–12% polyacrylamide gels with NuPAGE MES SDS running buffer with a prestained SeeBlue plus 2 protein molecular weight standard (Life Technologies, Sweden). Bands were transferred to nitrocellulose membranes using a Trans-Blot Turbo (Bio-Rad).

Primary TRIP13 antibodies SC-514285 and AB128178 (Santa Cruz Biotechnology, Santa Cruz, CA, and Abcam, UK, respectively) and horseradish peroxidase (HRP)-conjugated anti-rabbit or mouse HRP-IgG (W401B and W420B, respectively; Promega) were used for immunoblotting. All membranes were blocked using blocking buffer (5% w/v milk powder in Tris-buffered saline, with Tween 20, pH 8.0). Membranes were developed using Clarity Western ECL Chemiluminescent HRP-Substrate (Bio-Rad) according to the manufacturer’s recommendations. Chemiluminescence intensities were detected and quantified using a ChemiDoc XRS+ imaging system (Bio-Rad) with Image Lab software (Bio-Rad). CETSA curve band intensities were related to the intensities of the lowest temperature for the drug-exposed samples and control samples, respectively.

For the lysate study, the cells were collected and harvested as above and then directly lysed by three rounds of freeze–thaw in liquid nitrogen. The lysate was aliquoted, and the addition of 30 µM remdesivir was followed by incubation at room temperature for 15 min with gentle mixing. The suspensions were further aliquoted and subjected individually to a 12-temperature heat gradient between 37 and 63 °C for 3 min. Insoluble proteins were pelleted by centrifugation for 20 min at 20,000*g*; all subsequent protocols for detection were as described above.

## Results

We have applied the CETSA combined with quantitative LC-MS-based proteomics (CETSA MS) to profile compound-induced protein thermal stability changes for 22 compounds in intact HepG2 cells. The experiments were performed in compressed^21^ format (also known as one-pot format22). HepG2 cells were treated with 30 µM of each compound and incubated for 60 min at 37 °C in serum-free salt-based medium. After compound treatment, cell suspensions were divided into 12 aliquots, followed by heat shock treatment at 12 temperatures (44–66 °C) for 3 min. After heat treatment, for each incubation 12 differentially heated samples were pooled back, and aggregated proteins were removed by centrifugation. The resulting protein abundance in the soluble fraction corresponded to the area under the protein’s melting curve.^21^ The experiment was repeated to yield three biological replicates. Single-compound concentration and application of compressed (one-pot) experimental design allowed for the assessment of reliable protein stability changes at a relatively high throughput of ~6 h acquisition time per compound. The studied compounds will be incorporated in a larger (>200 compounds) initiative to establish CETSA-based molecular fingerprints of a diverse set of compounds.

Proteins were quantified via isobaric labeling LC-MS. The resulting dataset covers more than 8000 protein groups; of them, 5873 protein groups were reliably quantified in more than 17 out of 22 treatments, with at least two unique peptides (Suppl. Data 1).

In order to assess compound-induced protein thermal stability changes, for each treatment we compared log2-transformed and normalized intensities to the corresponding vehicle controls. For all 22 compounds tested, only 34 proteins were found to be significantly changed (stabilized or destabilized) upon treatment with at least one compound (see **Fig. 2**).

Remdesivir, ritonavir, baloxavir marboxil, and chloroquines demonstrate distinct proteome responses and form individual clusters in hierarchical clustering. The remaining compounds are represented in cluster 2.

Remdesivir, one of several nucleoside analogs in our panel, shows a clear hit for carboxylesterase 2 (CES2), which could be involved in the metabolism of the molecule. Although hydrolysis of the ester is reportedly by cathepsin A and carboxylesterase 1 (CES1),^23^ CES2 has high abundance in liver tissue. Also, acyl-coenzyme thioesterase 9 (ACOT9), as well as diphthine methyl ester synthase (DPH5), albeit less obviously, showed stability shift with remdesivir treatment; both of these proteins are known to bind esters, similar to the activity of CES2. Given that the activation of the prodrug includes an intracellular esterase hydrolysis step, an interaction is not surprising.

In contrast, and most notable from this study, is the destabilization of pachytene checkpoint protein 2 homolog (TRIP13). TRIP13 is a hexameric AAA+ ATPase and a key regulator in chromosome recombination and structural regulation, such as crossing over and DNA double-strand breaks.^24^ TRIP13 is essential in the spindle assembly checkpoint and is expressed in a number of human cancers, where its reduction has been linked with effects on proliferation and hence therapeutic benefit.^25^ It is plausible that remdesivir, in its fully synthesized triphosphate form, is competitive with endogenous ATP binding with TRIP13, disrupting or affecting multimerization with itself or downstream on the spindle assembly complex.

Interestingly, GS-441524, a metabolite of remdesivir, had no significant hits in this study. There could be multiple explanations for this, but in this case, it is established that unfavorable compound properties of GS-441524 result in limited cellular uptake, especially when a 60 min incubation protocol is considered. In our experience, the addition of nucleosides often has an impact on several proteins involved in cellular nucleoside homeostasis.

As apparent from **Figure 2**, the chloroquines (hydroxychloroquine, chloroquine, and chloroquine phosphate) comprise their own cluster. Despite having a clear function on the endosomal processes, the hits identified for hydroxychloroquine do not appear to follow an obvious pathway response, for example, vesicle proteins, vesicle lumen proteins (including endoplasmic reticulum Golgi), extracellular proteins, ion channels, and transporters. It is possible that the identified hits are the best representatives for the technique from their respective pathways. However, there are no known links between these targets to chloroquine or the hydroxy version.

Despite this, a common hit between all three chloroquine derivatives tested was choline kinase alpha (CHKA), which has a key role in phospholipid biosynthesis. Another common hit between the hydroxy and chloroquine phosphate forms is copine 1 (CPNE1), a calcium-dependent phospholipid binding protein that plays a role in calcium-mediated intracellular processes.^26^ Other significant hits are histamine *N*-methyltransferase (HNMT), epithelial cell adhesion molecule (EPCAM), and stathmin (STMN1).

As concluded earlier, both remdesivir and the chloroquines stand out as separate clusters, with no other antiviral compounds having similar response patterns. However, ritonavir, a protease inhibitor, and baloxavir marboxil also stand out with unique response patterns.

**Figure 1.**
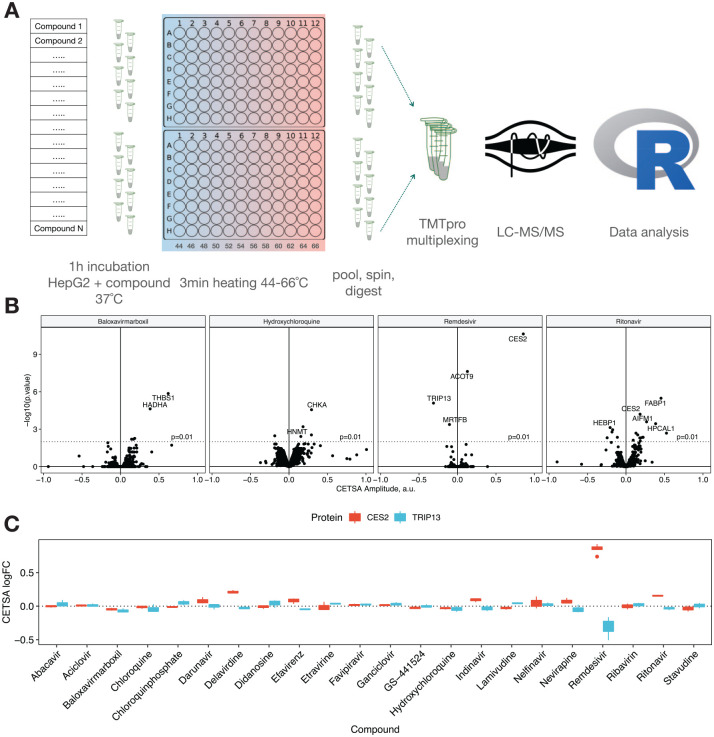
(**A**) Design of the experiment for CETSA MS profiling of 22 compounds in intact HepG2 cells. (**B**) Volcano plots summarizing proteins found to be stabilized/destabilized upon treatment of HepG2 cells with baloxavir marboxil (left), hydroxychloroquine, remdesivir, and ritonavir (right). (**C**) Box plot representation showing stability changes of TRIP13 and cocaine esterase CES2 relative to the vehicle control for all 22 compounds analyzed.

**Figure 2.**
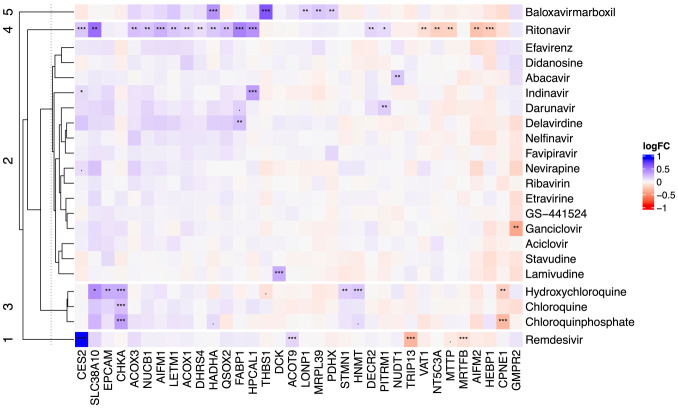
Heatmap of compound-induced protein thermal stability changes in HepG2 cells treated with different antiviral compounds. Proteins found to be significantly changed (*p* ≤ 0.01) in at least one compound are included in the plot.

Ritonavir induces many more significant shifts (**Figs. 1B and 2**) in comparison with the other protease inhibitors tested, darunavir, indinavir, and nelfinavir. Two of the hits, CES2 and DHRS4, could be implicated in the metabolism of ritonavir. The other stability-altered proteins may constitute a phenotype where lipid metabolism pathways are affected alongside Ca^2+^ and H^+^ ion balance. There is support in the literature for effects on lipid metabolism and the respiratory chain.^27,28^ Proteins involved in lipid metabolism that are thermally shifted by ritonavir include FABP1, MTTP, ACOX1, ACOX3, HADHA, DECR2, AIFM1, and AIFM2; the thiol modification protein QSOX2; ion channels LETM1 and SLC38A10; calcium and zinc binding proteins HPCAL1 and NUCB1; synaptic vesicle membrane protein VAT-1 homolog (VAT1); and finally, cytosolic 5′-nucleotidase 3A (NT5C3A), which dephosphorylates CMP and 7m-GMP. It should be noted, however, that most of the proteins shifted by ritonavir are also shifted, albeit to a lower extent, among the other protease inhibitors, as well as have a resemblance to the nonnucleoside inhibitor delavirdine. A possible explanation is that ritonavir has a faster cellular uptake or induction of cellular phenotypic effects, resulting in a significantly stronger shift in these patterns than other compounds.

Baloxavir marboxil, the antiviral medication for the treatment of influenzas A and B, has a quite distinct proteome stability alteration pattern. Baloxavir marboxil protein hits do not overlap with those of the other compounds. The proteins shifting include thrombospondin-1 (THBS1), an adhesive glycoprotein that mediates cell–cell and cell–matrix interactions; pyruvate dehydrogenase protein component (PDHX); mitochondrial ribosomal protein L39 (MRPL39); Lon protease (LONP1), an ATP-dependent serine protease; trifunctional enzyme subunit alpha (HADHA); and long-chain fatty acid-CoA ligase 1 (ACSL1). In our experience, such effects to the proteome are indicative of cellular stress response.

The remaining compounds either induce no shifts or do so for very few proteins. The latter make up cluster 2 in **Figure 2**, where the lack of a pronounced molecular fingerprint does not allow for further division into separate or unique groupings. Lamivudine treatment resulted in a stabilizing shift for DCK, which is known to be responsible for the intracellular phosphorylation of the drug,^29^ which provides confidence of cellular uptake. These data may well constitute useful information when taken within the context of further study.

Following the identification of TRIP13 as a destabilized target of remdesivir in the MS-coupled CETSA experiments, a follow-up CETSA study with immune-targeted detection of TRIP13 was employed. Here, the Western blot detection shows a destabilization of TRIP13 following remdesivir treatment in intact cells but not in a preprepared lysate experiment. The *T*_m_ value of TRIP13 differs substantially between the two matrices, with an approximately 8° lower *T*_m_ in the lysate sample. A reproducible remdesivir-induced destabilizing shift of 0.7° is observed in the intact sample, supporting the principal study finding from the CETSA MS datasets. Interestingly, there is no observed shift with remdesivir treatment in the lysate sample.

## Discussion

This study was intended to help us better understand any off-target effects of remdesivir and chloroquine as two prominently repurposed drugs for targeting SARS-CoV-2, with a view to identifying potential biological inroads for further investigation.

This is an intact cell study, and therefore it was conducted in a highly biological context. Thus, proteins exist at endogenous expression levels and environment. The relative amounts of analytes, nucleotides, and metabolites represent levels commensurate with healthy unmodified cultured cells. In the CETSA MS platform, we identify both stabilized and destabilized proteins after treatment with these drug molecules. A stabilizing shift is often attributed to a direct binding event. Similarly, a destabilizing shift can also be caused by a direct binding event if the molecular interaction causes the target to be less thermodynamically favorable. Additionally, destabilizing events can be caused by the usurping of a native substrate or the removal of a complex of protein–protein interactions as a secondary downstream effect.

The host targets identified represent a wide variety of biological processes. It is important to note that these data are included in this study to offer an unrevised perspective at potential off-targets. A thorough understanding of the relevance to viral infection is a significant undertaking and well beyond the scope and timelines of this study. The term “off-targets” or “unintended targets” is employed here, meaning not the primary target, although it can also be the case that an interaction with a host protein is essential for the efficacy of the drug, as is the case with lamivudine and DCK. In this light, there are other targets involved in nucleotide regulation, such as RNR, SAMHD1, and ADK in particular, that we would have expected the cellular presence of GS-441524 and other nucleotide analogs to have affected. In contrast, these regulatory proteins were not identified as hits, which is a surprising outcome given that, in our experience, ADK is known to shift upon binding of a substrate.

There are no previous studies using CETSA MS to comparatively analyze a panel of antiviral compounds. Given that the primary purpose of the majority of these drugs is to interact with or inhibit viral proteins, there was no expectation that common host targets would be identified.

In contrast, the chloroquine molecules are known to have substantial effects on the endosomal compartment and the expectation was significant; however, broad shifts in these samples were not observed. Aside from the described shifts, the bulk of cluster 2 represented less defined changes to a broad range of biological activities, not allowing for a definitive molecular fingerprint to be elucidated.

This study was designed to identify previously unidentified proteins that could have critical importance for the reported activity of remdesivir and other compounds in the context of COVID-19. The inclusion of a panel of molecules allows for cross-comparison against hits specific to one molecule, which facilitated the novel finding that remdesivir uniquely destabilizes TRIP13.

The function of TRIP13 does not lend itself to being an obvious benefit or hinderance to viral infection, as would be considered by a protein with known host innate viral immunity activity. But the fact that it interacts with nucleotides and forms a homohexamer that, if diminished, removes activity gives credence to the possibility that interaction with remdesivir may in fact be tangible.^30^

**Figure 3.**
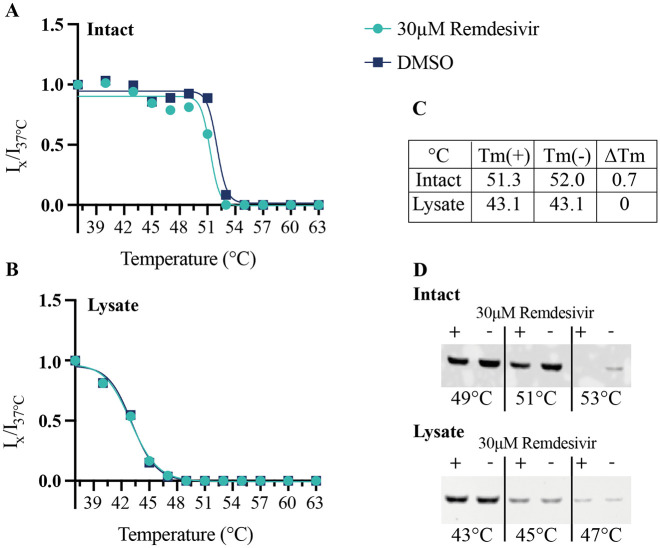
Western blot CETSA analysis of remdesivir destabilization of TRIP13 Band intensities of TRIP13 melt curves ± 30 µM remdesivir on (**A**) intact HepG2 cells and (**B**) lysed HepG2 cells. (**C**) Table representing apparent *T*_m_ values and delta *T*_m_ shift. (**D**) Western blots representing a selection of temperatures form the complete melt curves for both intact and lysate samples. Band corresponding to correct molecular weight for TRIP13.

It is distinctly clear (**Fig. 3**) that the *T*_m_ value of TRIP13 in lysate is substantially lower than that of intact cells; this is likely due to the bulk dilution effect of lysing cells into a buffered solution. Nucleotides, metabolites, and proteins that could form homo- or heterocomplexes are substantially diluted. Given that the biology of the target involves the formation of higher-order complexes, this difference in *T*_m_ could be attributed to the disruption of these complexes combined with the dilution of ATP after lysis. Interestingly, unlike the intact sample, the lysate sample has no apparent shift after the incubation with remdesivir. Even though the incubation step is shorter and at a lower temperature in the lysate sample, we proffer that remdesivir itself has not been converted to its active form by proteins that were hits in the intact MS CETSA experiments, such as CES2. While these proteins, essential for the activation of remdesivir, are likely present in the lysate sample, the concentration would be significantly reduced. A useful study would be to test other forms of remdesivir to elucidate whether the active form of the drug or one of the several activation steps are responsible for the TRIP13 destabilizing activity.

Further in vitro biophysical investigation probing the interaction could elucidate evidence into the role of TRIP13 in remdesivir therapy. The functional relevance of such an interaction in the context of virus-infected tissue could yield crucial information as to whether its potential off-target behavior is tolerable, beneficial, or indeed a hindrance to the molecule’s efficacy against SARS-CoV-2. Also, remdesivir may constitute a starting point for developing antitumor therapies directed against TRIP13.

This study has highlighted the power of utilizing unbiased whole-proteome approaches and the information that can be rapidly gained from describing proteome-wide target engagement of drug molecules.
